# Integrated Analysis of the Fecal Metagenome and Metabolome in Bladder Cancer in a Chinese Population

**DOI:** 10.3390/genes13111967

**Published:** 2022-10-28

**Authors:** Chuan Qin, Zhenghao Chen, Rui Cao, Mingjun Shi, Ye Tian

**Affiliations:** Department of Urology, Beijing Friendship Hospital, Capital Medical University, Beijing 100068, China

**Keywords:** metagenomics, metabolomics, gut microbiome, omics integration, bladder cancer

## Abstract

Bladder cancer (BLCA) is a common malignancy of the urinary system. The gut microbiome produces various metabolites that play functional roles in tumorigenesis and tumor progression. However, the integrative analysis of the gut microbiome and metabolome in BLCA has still been lacking. Thus, the aim of this study was to identify microbial and functional characteristics and metabolites in BLCA in a Chinese population. Metagenomics, targeted metabolomics, bioinformatics, and integrative analysis were used in fecal samples of BLCA patients and healthy individuals. We found gut microbiomes were significantly dysregulated in BLCA patients, including *Bifidobacterium*, *Lactobacillus*, *Streptococcus*, *Blautia*, and *Eubacterium*. We also found 11Z-eicosenoic acid, 3-methoxytyrosine, abrine, aniline-2-sulfonate, arachidic acid, conjugated linoleic acids, elaidic acid, glycylleucine, glycylproline, leucyl-glycine, linoelaidic acid, linoleic acid, nicotinamide hypoxanthine dinucleotide, oleic acid, petroselinic acid, and ricinoleic acid to be significantly decreased, while cholesterol sulfate was significantly increased in BLCA patients. Integration of metagenomics and metabolomics revealed interactions between gut microbiota and metabolites and the host. We identified the alterations of gut microbiomes and metabolites in BLCA in a Chinese population. Moreover, we preliminarily revealed the associations between specific gut microbiomes and metabolites. These findings determined potential causative links among gut dysbiosis, dysregulated metabolites, and BLCA.

## 1. Introduction

Bladder cancer (BLCA) is one of the most common malignancies of the urinary system. More than 570,000 patients are newly diagnosed, and more than 210,000 deaths are reported each year worldwide [1]. In 2015, the morbidity rate of BLCA in China was 5.80 per 100,000 and the mortality rate of BC in China was 2.37 per 100,000 [2]. In China, BLCA causes a serious social and economic burden. On the basis of the invasion of detrusor muscle, BLCA can be divided into non-muscle-invasive BLCA (NMIBC) and muscle-invasive BLCA (MIBC) [3]. NMIBC are more inclined to tumor recurrence (approximately 50–70%) and progression (approximately 15%), while MIBC often has a high risk of tumor metastasis and a low overall survival rate. For pathological types of BLCA, urothelial bladder carcinoma (UBC), previously often described as transitional cell cancer, is the predominant histological type (more than 90%) [4]. The occurrence and progression of BLCA include multifactorial and multistep pathophysiological processes, and the regulatory mechanisms are not fully understood. Therefore, it is essential to explore the relationship between related molecules and the occurrence and progression of BLCA from various perspectives.

As we know, mammals have a large and diverse symbiotic microbial community in the intestine. The total mass of microorganisms in the intestine of a normal adult can reach 1–1.5 kg, which contains up to 1 × 10^14^ bacteria [5]. Such a large micro community of organisms constitute an extremely complex collective, namely, the gut microbiota. The gut microbiome and the host interact with each other and gradually form a symbiotic homeostatic system, which plays an extremely important role in the growth, development, nutrient metabolism, and immune homeostasis of the host [6]. The metabolism, homeostasis, and immune system of gut are maintained with the help of gut microbiome. When their populations or living conditions are “dysregulated”, it may lead to metabolic abnormalities; inflammation; and other diseases, such as obesity [7], type 2 diabetes mellitus [8], and food allergies [9]. Moreover, an increasing number of studies have shown that the gut microbiome plays an important role in tumorigenesis and tumor progression, such as breast cancer [10], prostate cancer [11], and colorectal cancer [12]. Recently, numerous studies have shown that the gut microbiome produces a variety of metabolites that have different functional roles in tumorigenesis and tumor progression, such as short-chain fatty acids (SCFAs) [13], secondary bile acids [14], and polyamines [15]. Many researchers have been exploring the gut microbiota–metabolites–disease axis, which results in a pro-tumorigenic phenotype in multiple solid tumor types. For instance, IGF1 production stimulated by SCFAs from gut microbes influenced the growth of prostate cancer via activating local prostate MAPK and PI3K signaling, indicating the existence of a gut microbiota–IGF1–prostate axis [13]. In another case, gut microbiota promoted myeloid leukemia progression via the downregulation of butyrate, which can repair intestinal barrier damage and inhibit LPS absorption in vivo [16].

With the rapid development of metagenomics and metabolomics, the potential relationship of gut microbiomes and metabolites and cancer could be determined comprehensively. However, the integrative analysis of fecal metagenomics and metabolomics in BLCA was still lacking. In the current work, we used quantitative metagenomic and metabolomic analysis to identify genic, microbial, and functional characteristics and metabolites in BLCA. These findings were expected to determine a potential causative link among gut dysbiosis, dysregulated metabolites, and BLCA. 

## 2. Materials and Methods

### 2.1. Subjects and Sample Collection

A total of 32 BLCA patients (EXP group) were recruited at the Department of Urology of Beijing Friendship Hospital affiliated with Capital Medical University (Beijing, China) in 2021–2022. The fecal samples of BLCA patients were collected before surgery. The inclusion criteria for patient selection included: (1) being ethnically Han; (2) being without radiotherapy, chemotherapy, or other adjuvant treatments; (3) age > 18 years; (4) postoperative pathological diagnosis of UBC with or without lymph node metastasis and distant metastasis; (5) normal intelligence; (6) satisfactory pulmonary, cardiac, liver, renal, and bone marrow functions. Exclusion criteria for the patients were as follows: (1) age under 18; (2) treatment with antibiotics or anti-inflammatory agents 1 month before sampling; (3) being vegetarian; (4) being pregnant; (5) having a history of other tumors or inflammatory bowel disease; (6) a postoperative pathological diagnosis of non-UBC; (7) having autoimmune diseases; (8) declining to participate after reading the informed consent form. A total of 15 gender-, age-, and body-weight-matched healthy controls (CON group) were recruited for this study. This study was approved by the Ethics Committee of Beijing Friendship Hospital affiliated with Capital Medical University and was conducted in accordance with the Declaration of Helsinki ethical standards. Informed consent was obtained from all participants and healthy individuals. Fresh fecal samples were collected from each subject and were stored at a −80 °C freezer immediately.

### 2.2. Experimental Procedures of Metagenomic Sequencing

#### 2.2.1. DNA Extraction and Sample Testing

Total DNA was extracted from the fecal samples of all BLCA patients and healthy individuals using a Magnetic Soil and Stool DNA Kit (DP712, TIANGEN, Beijing, China) on the basis of the manufacturer’s instructions. Next, DNA degradation and potential contamination was detected on 1% agarose gels. Then, DNA concentration was measured using a Qubit^®^ dsDNA Assay Kit in Qubit^®^ 2.0 Flurometer (Life Technologies, Carlsbad, CA, USA). DNA contents with OD values between 1.8 and 2.0 were used for next step. Only qualified samples were used for library construction.

#### 2.2.2. Library Construction

A total amount of 1 μg DNA per sample was used as input material for the DNA sample preparations. Sequencing libraries were generated using a NEBNext^®^ Ultra ™ DNA Library Prep Kit for Illumina (NEB, Ipswich, MA, USA), according to the instructions. Next, index codes were added to attribute sequences to each sample. Then, the DNA sample was fragmented by sonication to a size of 350 bp. Following this, DNA fragments were end-polished, A-tailed, and ligated with the full-length adaptor for Illumina sequencing with further PCR amplification. PCR products were purified (AMPure XP system), and libraries were analyzed for size distribution by an Agilent 2100 Bioanalyzer and quantified using real-time PCR (Bio-Rad CFX96). A total of 35 cycles were set for the PCR test.

#### 2.2.3. Sequencing

The clustering of the index-coded samples was performed on a cBot Cluster Generation System according to the instructions. After cluster generation, the library preparations were sequenced on an Illumina Novaseq 6000 (or MGISEQ-T7), and 150 bp paired-end reads were generated.

### 2.3. Data Analysis of Metagenomic Project

#### 2.3.1. Data Pretreatment and Quality Control

For quality control, default parameters of fastp (https://github.com/OpenGene/fastp, accessed on 10 May 2022) software was used to pre-process the raw data obtained from Illumina sequencing platforms to obtain clean data for subsequent analysis. The specific processing steps were as follows: (1) we removed the reads which contain adapter; (2) we removed the reads for which the N (N means that the base information cannot be determined) ratio was greater than 10%; (3) we removed the reads with low quality (the number of bases with Q ≤ 15 accounted for more than 50% of the reads). Last, considering the possibility of host pollution may exist in samples, clean data needed to be blasted to the host database, using the default Bowtie2.2.4 software (version 2.2.4, http://bowtie-bio.sourceforge.net/bowtie2/index.shtml, accessed on 1 January 2020) to filter out reads that may have originated from the host [17].

#### 2.3.2. Metagenome Assembly

SOAPdenovo software (V2.04, http://soap.genomics.org.cn/soapdenovo.html, accessed on 13 May 2022) was used to perform the assembly and analysis of clean data for fecal samples [18]. Then, the assembled Scaftigs from N connection were interrupted, and the Scaftigs without N were obtained. All samples’ clean data were compared to each scaffold by Bowtie2.2.4 software to acquire the PE reads [19]. All the reads that were not used in the forward step of all samples were combined, and following this, the software of SOAPdenovo was used for mixed assembly with the parameters. The Scaftigs were obtained, and the fragments shorter than 500 bp in all Scaftigs for statistical analysis were filtered.

### 2.4. Gene Prediction and Abundance Analysis

MetaGeneMark (V2.10, http://topaz.gatech.edu/GeneMark/, accessed on 17 May 2022) software was used to predict the ORF for the Scaftigs (>500 bp) assembled. The length information shorter than 100 nt [20] from the predicted results with default parameters were filtered and excluded. For ORF predicting, CD-HIT software (V4.5.8, http://www.bioinformatics.org/cd-hit/, accessed on 17 May 2022) [21] was used for de-redundancy and for obtaining the unique initial gene catalogue. Then, the clean data of each sample were mapped to initial gene catalogue using Bowtie2.2.4, and the number of reads of the genes mapped in each sample was calculated. The gene for which the number of reads < 2 in each sample was filtered and excluded. The gene catalogue (unigenes) was obtained for subsequent analysis. Unigene is an acronym for Universal Gene, which stands for widely used gene database, forming a non-redundant gene database through a computerized collection of identical loci. On the basis of the number of mapped reads and the length of gene, the abundance information of each gene in each sample was calculated. On the basis of the abundance information of each gene in each sample in the gene catalogue, general information statistics, core-pan gene analysis, correlation analysis of samples, and Venn analysis were performed.

### 2.5. Taxonomy Prediction

First, DIAMOND software (V0.9.9, https://github.com/bbuchfink/diamond/, accessed on 20 May 2022) was used to compare the unigenes with bacterial, fungal, archaeal, and viral sequences extracted from NCBI’s NR database (version 2018-01-02, https://www.ncbi.nlm.nih.gov/, accessed on 20 May 2022). Second was the comparison of each sequence, the results of which the e values ≤ 1 × 10^−5^ were analyzed by the LCA algorithm to determine the species annotation information for the sequence. On the basis of the LCA annotation results and gene abundance, we obtained the abundance information and gene numbers of each sample in each taxonomy hierarchy (kingdom, phylum, class, order, family, genus, species). On the basis of the abundance at each taxonomic level, Krona analysis, generation situation of relative abundance, and the abundance cluster heat map were drawn. Pareto-scaled principal component analysis (PCA, R ade4 package, Version 2.15.3) and NMDS (R vegan package, Version 2.15.3) were used to perform dimension reduced analysis. The difference between groups was tested by Anosim analysis (R vegan package, Version 2.15.3). Metastats and LEfSe analysis were used to identify the different species between groups. A permutation test between groups was performed using Metastats analysis for each taxonomy. After obtaining the *p*-value, Benjamini and Hochberg false discovery rate (FDR) was used to acquire the q-value [22]. *p*-value < 0.05 was considered statistically significant, and the q-value was calculated to evaluate the FDR for correction of multiple comparisons. LEfSe analysis was conducted by LEfSe software (the default LDA score is 3). Finally, random forest (R pROC and random forest packages, version 2.15.3) was used to construct a random forest model. Significant species were screened out by mean decrease accuracy and mean decrease gin, and each model (default 10 times) was cross-validated.

### 2.6. Functional Database Annotations

DIAMOND software (V0.9.9, https://github.com/bbuchfink/diamond/, accessed on 23 May 2022) was used to compare the unigenes with functional databases, including KEGG (Version 2018-01-01, http://www.kegg.jp/kegg/, accessed on 23 May 2022), eggNOG (Version 4.5, http://eggnogdb.embl.de/#/app/home, accessed on 23 May 2022), and CAZy (Version 201801, http://www.cazy.org/, accessed on 24 May 2022). For each sequence’s result, the Best Blast Hit was used for subsequent analysis. 

### 2.7. Experimental Procedures of Targeted Metabolomics

#### 2.7.1. Metabolite Extractions

First, 800 μL of cold methanol/acetonitrile/water (2:2:1, *v*/*v*) extraction solvent was added to 100 mg sample, and the mixture was adequately vortexed. Stock solutions of stable-isotope internal standards were added to the extraction solvent simultaneously. Then, the samples were under vigorous shaking and incubated on ice, and following this, were centrifuged at 14,000× *g* for 20 min at 4 °C, whereafter the supernatant was collected and flowed through a 96-well protein precipitation plate, and then the elution was collected and dried in a vacuum centrifuge at 4 °C. For LC–MS analysis, the samples were re-dissolved in 100 μL acetonitrile/water (1:1, *v*/*v*) solvent and centrifuged at 14,000× *g* at 4 °C for 15 min, and following this, the supernatant was injected.

#### 2.7.2. LC–MS/MS Analysis

Analyses were performed using an UHPLC (1290 Infinity LC, Agilent Technologies, Santa Clara, CA, USA) coupled to a QTRAP MS (6500+, Sciex, Framingham, MA, USA). The analytes were separated on HILIC (Waters UPLC BEH Amide column, 2.1 mm × 100 mm, 1.7 µm) and C18 columns (Waters UPLC BEH C18-2.1 × 100 mm, 1.7 μm). For HILIC separation, the column temperature was set at 35 °C, and the injection volume was 2 μL. A gradient was then initiated at a flow rate of 300 μL/min. For RPLC separation, the column temperature was set at 40 °C, and the injection volume was 2 μL. A gradient was then initiated at a flow rate of 400 μL/min. The sample was placed at 4 °C during the whole analysis process. Next, 6500 + QTRAP (AB SCIEX) was performed in positive and negative switch modes. The ESI-positive source conditions were as follows: source temperature: 580 °C; Ion Source Gas1 (GS1): 45; Ion Source Gas2 (GS2): 60; Curtain Gas (CUR): 35; IonSpray Voltage (IS): +4500 V. The ESI-negative source conditions were as follows: source temperature: 580 °C; Ion Source Gas1 (GS1): 45; Ion Source Gas2 (GS2): 60; Curtain Gas (CUR): 35; IonSpray Voltage (IS): −4500 V. The MRM method was used for mass spectrometry quantitative data acquisition. Polled quality control samples were set in the sample queue to evaluate the stability and repeatability of the system.

#### 2.7.3. Data Processing

Multi-Quant or Analyst was used for quantitative data processing. The QCs were processed together with the biological samples. Metabolites in QCs with coefficient of variation less than 30% were denoted as reproducible measurements.

#### 2.7.4. Statistical Analysis

After sum-normalization, the processed data were uploaded into before importing into SIMCA-P (version 14.1, Umetrics, Umea, Sweden), where it was subjected to multivariate data analysis, including PCA and orthogonal partial least-squares discriminant analysis (OPLS-DA). The sevenfold cross-validation and response permutation testing were used to evaluate the robustness of the model. The variable importance value (VIP) in the projection of each variable in the OPLS-DA model was calculated to indicate its contribution to the classification. Significance was determined using an unpaired Student’s *t* test. A *p*-value < 0.05 was considered statistically significant.

### 2.8. Integrated Analysis of Metagenomics and Metabolomics

After metagenomics and metabolomics profiling, the top 11 metabolites with the most significant differences (metabolites with top 11 VIP values and *p*-value < 0.05 with qualitative information) and top 18 microbiomes with the most significant differences (microbiomes with top 18 VIP values and *p*-value < 0.05 with qualitative information) were selected for the integrated analysis. First, PCA and PCoA were used to perform the multi-omics downscaled comparative analysis. Then, multiple direct gradient analysis was performed using RDA. RDA, based on a linear model, was used to reflect the relationship between gut microbiomes and significant fecal metabolites. Then, a weighted network was built by computing the Spearman analysis. Moreover, the correlation coefficient between metabolites and microbiomes were shown as a heat map. A *p*-value < 0.05 was considered statistically significant in this study.

## 3. Results

### 3.1. Patient Characterization

In the present study, a total of 32 BLCA patients (EXP group) and 15 normal healthy individuals (CON group) were recruited. The age for BLCA patients and healthy individuals were 65.06 ± 9.277 and 60.60 ± 13.96, respectively. For gender, there were 21 males and 11 females among BLCA patients, and 10 males and 5 females among healthy individuals. Notably, there were no statistical significances in terms of age and gender of the two groups. The postoperative pathology revealed that EXP group includes 7 low-grade patients and 25 high-grade patients. Moreover, there were 6 MIBC (T2 or above) and 26 NMIBC (Ta and T1) in the EXP group. In particular, to reduce disturbing factors, UBC was the only pathological type for all patients after selection.

### 3.2. Gene Number Differences and Correlation Analysis

**Figure 1:**
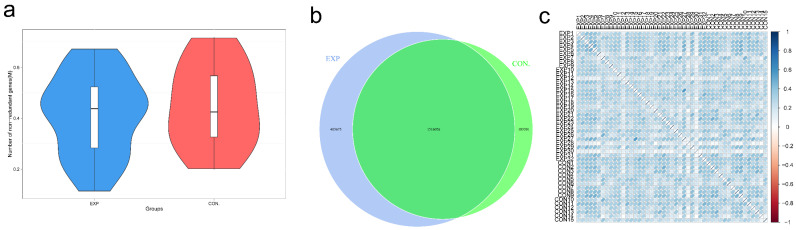
Gene number differences and correlation analysis (BLCA patients, EXP group; healthy individuals, CON group). (**a**) Box plot of gene number between groups. The horizontal coordinate stands for each group, and the vertical coordinate stands for the gene numbers. (**b**) Venn graph. Each circle represents a group. The number in overlapping circles represents the gene numbers shared between groups; the number in non-overlapping circles represents the number of genes unique to the group. (**c**) Sample correlation coefficient heat map. Different colors represent the level of Spearman correlation coefficient. The leftward bias of the ellipse indicates that the correlation coefficient is positive, and the rightward bias is negative.

To identify the total number of genes that differed between groups, box plots of gene numbers between EXP and CON group were drawn. As shown in Figure 1a, total gene numbers in the EXP group were slightly more than the CON group. To examine the distribution of gene numbers between the two groups, a Venn graph was drawn. As shown in Figure 1b, there were 1,516,026 genes commonly detected in the two groups, 485,675 genes specifically detected in the EXP group, and 183,301 genes specifically detected in the CON group. Moreover, we performed the correlation of gene abundance between samples. As shown in Figure 1c, different colors represented the level of Spearman correlation coefficient. The similarity of gene abundance patterns between samples was relatively high.

### 3.3. Relative Abundance Analysis of Gut Microbiota

**Figure 2:**
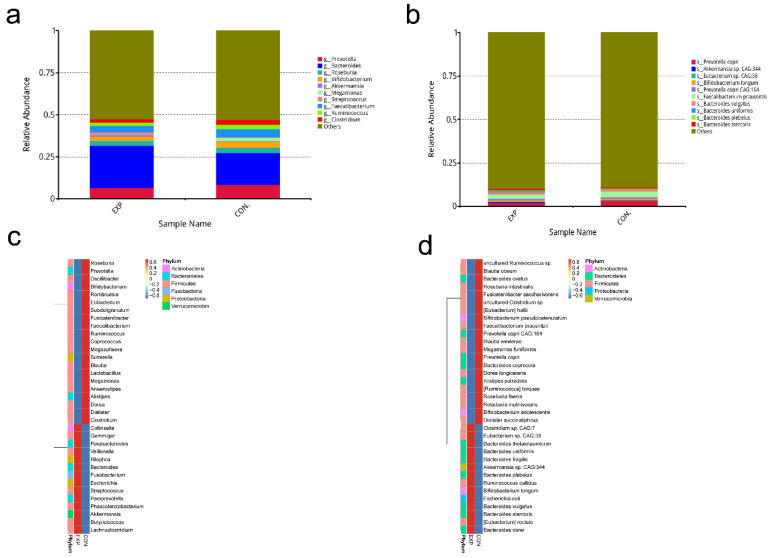
Relative abundance analysis of gut microbiota (BLCA patients, EXP group; healthy individuals, CON group). (**a**) At the genus level, the top 10 microbiomes with maximum relative abundance in two groups are shown. (**b**) At the species level, the top 10 microbiomes with maximum relative abundance in the two groups are shown. The number in non-overlapping circles represents the number of genes unique to the group. (**c**) Heat map of the top 35 microbiomes of abundance ranking in the two groups at genus level. (**d**) Heat map of the top 35 microbiomes of abundance ranking in the two groups at the species level.

Histograms were plotted for each group, corresponding to the annotation results at genus and species levels. At the genus level (Figure 2a), the results showed *Prevotella*, *Bacteroides*, *Roseburia*, *Bifidobacterium*, *Akkermansia*, *Megamonas*, *Streptococcus*, *Faecalibacterium*, *Ruminococcus*, and *Clostridium* were the top 10 microbiomes with maximum relative abundance in two groups. At the species level (Figure 2b), the top 10 microbiomes with maximum relative abundance in the two groups were shown. On the basis of the relative abundance at different taxonomic levels, we selected the abundance information of the top 35 microbiomes in the two groups to create a heat map. At the genus level (Figure 2c), 21 microbiomes had higher abundance in the CON group, including *Bifidobacterium* and *Lactobacillus*. However, 14 microbiomes had higher abundance in the EXP group, including *Streptococcus* and *Escherichia*. At the species level (Figure 2d), 35 microbiomes in two groups under their genus were shown, which are listed in Table S1. Moreover, the heat maps also showed the phylum of these top 35 microbiomes (*Actinobacteria*, *Bacteroidetes*, *Firmicutes*, *Proteobacteria*, *Verrucomicrobia*).

### 3.4. Dimension-Reducing Analysis Based on Species Abundance

**Figure 3:**
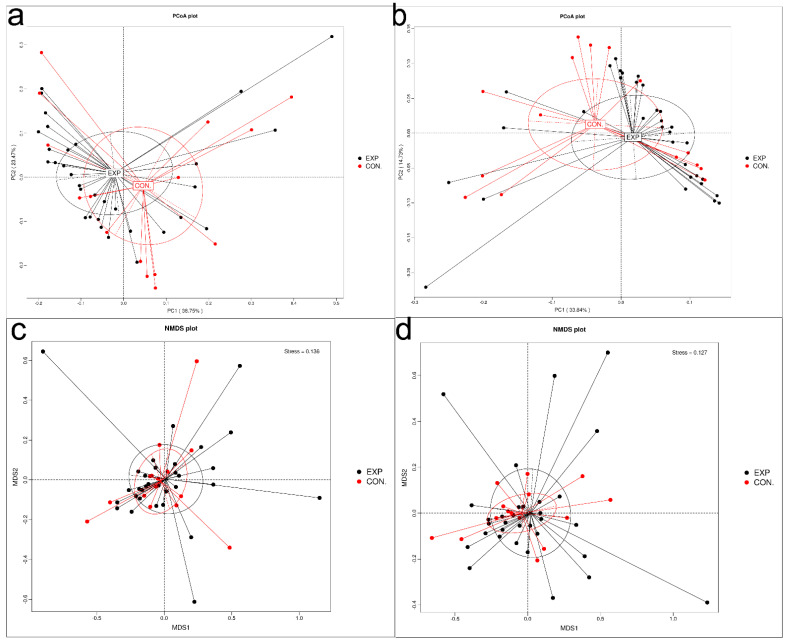
Dimension-reducing analysis based on species abundance (BLCA patients, EXP group; healthy individuals, CON group). Each point in the graph indicates a sample, and samples from the same group were represented using the same color. The distance between points indicated the degree of difference. (**a**) At the genus level, PCoA is shown. (**b**) At the species level, PCoA is shown. (**c**) At the genus level, NMDS is shown. Stress point = 0.136. (**d**) At the species level, NMDS is shown. Stress point = 0.127.

First, principal co-ordinates analysis (PCoA) was used to extract the most dominant elements and structures from multidimensional data by a series of eigenvalues and through eigenvector sorting. We performed PCoA based on Bray–Curtis distance and selected the combination of principal coordinates with the highest contribution rate for graphical presentation. As shown in Figure 3a,b, a BLCA microbial dysbiosis was revealed because overlap was found only in part with taxonomic composition in EXP and CON at genus and species level. Next, non-metric multi-dimensional scaling (NMDS) analysis was performed to overcome the shortcomings of linear models to better reflect the non-linear structure of ecological data. As shown in Figure 3c,d, there were some differences of species composition that were based on this analysis for at genus and species levels (stress points = 0.136 and 0.127, respectively). Stress points less than 0.2 indicated that NMDS analysis had reliability.

### 3.5. LEfSe and Metastat Analysis of Species Differing between Groups

**Figure 4:**
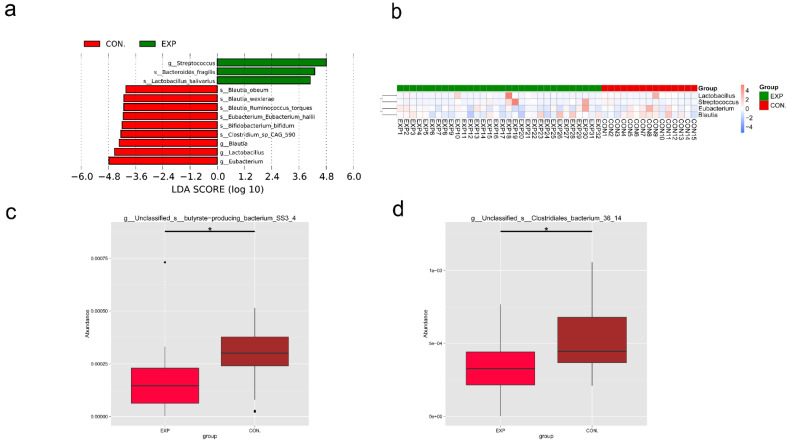
LEfSe and Metastat analysis of species differing between groups (BLCA patients, EXP group; healthy individuals, CON group). (**a**) Species with an LDA score greater than a set value (set to 4 by default) are shown. The length of the bar graph represents the effect size of the differential species (i.e., the LDA score). (**b**) Cluster heat map based on differential species. The clustering tree on the left is the species clustering tree, and the values corresponding to the middle heat map are the Z values obtained by normalizing the relative abundance of species in each row. (**c**) Metastat analysis of *butyrate-producing bacterium SS3_4*. * indicates q-value < 0.05. (**d**) A Metastat analysis of *Clostridiales bacterium 36_14*. * indicates q-value < 0.05.

To screen for species with significant differences between groups in biomarkers, we first detected species with differences between groups by means of rank sum test and achieved dimensionality reduction by LDA (linear discriminant analysis); following this, we evaluated the effect size of the difference species. Then, the LDA score was obtained. As shown in Figure 4a, species with LDA scores greater than a set value (set to 4 by default) were revealed, which could be considered biomarkers that are statistically different between groups, including *Streptococcus*, *Blautia*, *Bifidobacterium*, *Lactobacillus*, and *Eubacterium*. The length of the bar chart represented the effect size of the differing species. As shown in Figure 4b, on the basis of the abundance of species that differed between groups as screened by LEfSe above, the differential species at the genus level were selected to draw a cluster heat map to reflect the distribution of these differential species across samples, including *Streptococcus*, *Blautia*, *Lactobacillus*, and *Eubacterium*. Next, we used the Metastats method to process the species abundance data between the EXP and CON groups. By correcting for the *p*-value, the q-value was obtained. As shown in Figure 4c,d, we found the abundance of *butyrate-producing bacterium SS3_4* and *Clostridiales bacterium 36_14* was significantly reduced in the EXP group compared to the CON group at the species level (q-value < 0.05).

### 3.6. Functional Characterization of Gut Microbiota

**Figure 5:**
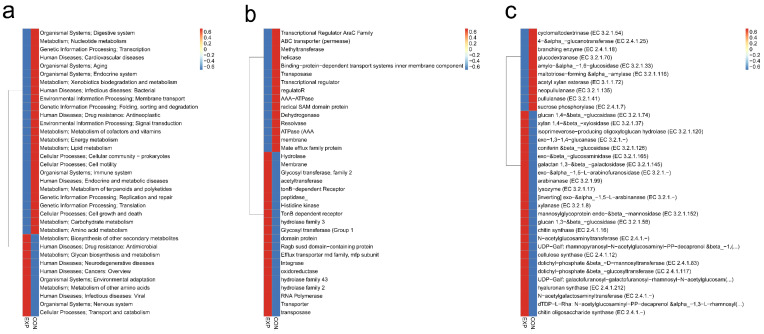
Functional characterization of gut microbiota (BLCA patients, EXP group; healthy individuals, CON group). (**a**) Distributions of relative abundances of KEGG pathway categories in EXP and CON groups. (**b**) Distributions of relative abundances of eggNOG pathway categories in EXP and CON groups. (**c**) Distributions of relative abundances of CAZy pathway categories in EXP and CON groups.

We selected the top 35 functions in terms of abundance and their abundance information in each sample on the basis of the function annotations and abundance information of all samples in each database in order to create a heat map and clustered them at the level of functional differences. On the basis of the KEGG pathway (Kyoto Encyclopedia of Genes and Genomes, Figure 5a), eggNOG (Evolutionary Genealogy of Genes: Non-Supervised Orthologous Groups, Figure 5b), and CAZy (Carbohydrate-Active Enzymes Database, Figure 5c) comparisons, we found that gut microbiomes in the EXP and CON groups were abundant in various pathways. The detailed information is shown in Table S2. These results suggest that dysbiosis microbiota in BLCA patients could be involved in many pathophysiological activities of the human body.

### 3.7. Changes of Gut-Microbiome-Associated Metabolites in BLCA Patients

**Figure 6:**
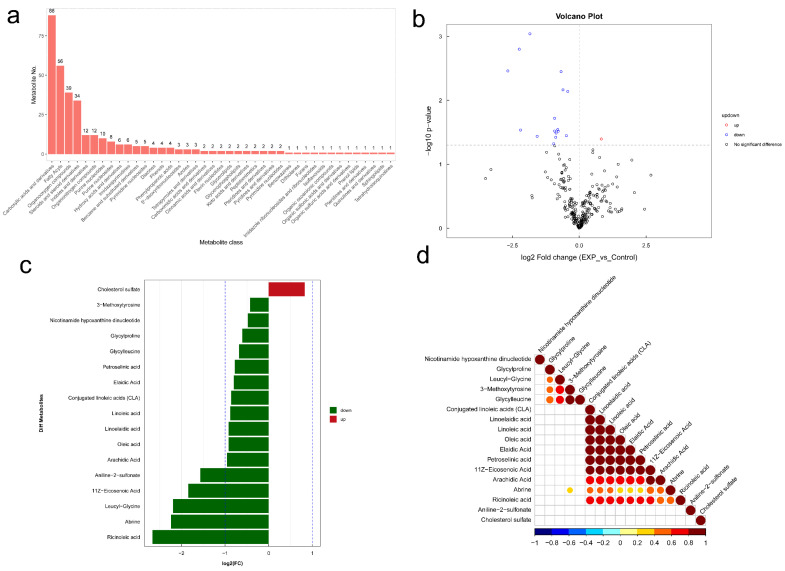
Changes of gut-microbiome-associated metabolites in BLCA patients (BLCA patients, EXP group; healthy individuals, CON group). (**a**) Quantitative statistics of the identified metabolites in each chemical classification. Each bar in the figure stands for a different chemical classification attribution entry. (**b**) Volcano plot was used to visualize the differential analysis for all metabolites detected. The horizontal coordinate was the log2 value of the fold change (FC), and the vertical coordinate was the log10 value of the significant *p*-value. Significantly dysregulated metabolites: metabolites meeting FC > 1, *p*-value < 0.05, are shown in red; metabolites meeting FC < 1, *p*-value < 0.05 are shown in blue. Non-significantly dysregulated metabolites are shown in black. (**c**) The fold change of identified metabolites with statistical significances are shown. Upregulation of differential metabolites are shown in red. Downregulation of differential metabolites are shown in green. (**d**) Heatmap of correlation analysis for these significantly dysregulated metabolites. Red indicates positive correlation, blue indicates negative correlation, and white indicates non-significant correlation. The size of the dot is related to the significance of the correlation.

In this section, targeted metabolomics was used to perform the qualitative and quantitative analysis of relevant metabolites in EXP and CON groups. The results revealed a total of 348 fecal metabolites identified after positive and negative ion switching detection. All metabolites were classified according to their chemical taxonomy attribution (CTA) information. The number of metabolites for each CTA is shown in Figure 6a. As a result, carboxylic acids and derivatives, fatty acyls, organooxygen compounds, steroids and steroid derivatives, indoles and derivatives, organonitrogen compounds, and purine nucleotides were found to have more than 10 metabolites. Next, a volcano plot was used to visualize the differential analysis for all metabolites detected. As shown in Figure 6b, a total of 18 metabolites showed consistent variances with statistical significances between the two groups (cutoff: fold change > 1 and *p*-value < 0.05). Among them, compared to the CON group, 17 metabolites were decreased, while only 1 metabolite was increased in the EXP group. In order to visualize the fold change of identified metabolites with statistical significances, a histogram was drawn, as shown in Figure 6c. As a result, compared to the CON group, 11Z-eicosenoic acid, 3-methoxytyrosine, abrine, aniline-2-sulfonate, arachidic acid, conjugated linoleic acids, elaidic acid, glycylleucine, glycylproline, leucyl-glycine, linoelaidic acid, linoleic acid, nicotinamide hypoxanthine dinucleotide, oleic acid, petroselinic acid, and ricinoleic acid were reduced, while cholesterol sulfate was increased in BLCA patients. Then, correlation analysis was performed to measure the metabolic proximities in significantly dysregulated metabolites (*p*-value < 0.05). As shown in Figure 6d, these dysregulated metabolites might have some expression correlations that indicated that they may be involved in several biological processes together, namely, functional relevance. Last, according to the enrichment score of KEGG analysis, biosynthesis of unsaturated fatty acids (hsa01040, *p*-value < 0.05) and linoleic acid metabolism (hsa00591, *p*-value < 0.05) were statistically significant pathways for these dysregulated metabolites. The KEGG pathway annotation results and maps are shown in Table S3 and Figure S1.

### 3.8. Integrative Analysis of Metagenomics and Metabolomics

**Figure 7:**
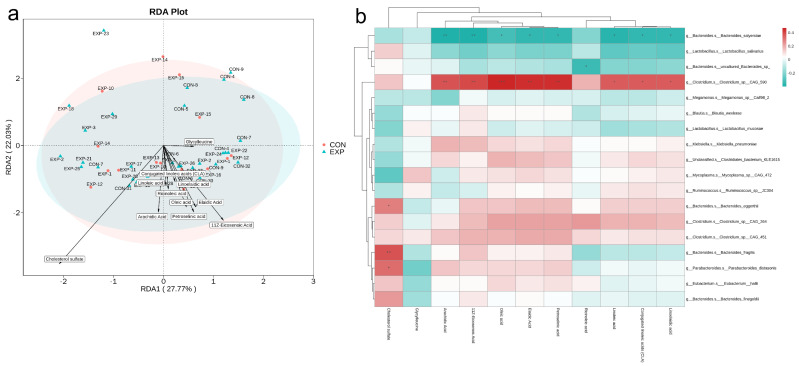
Integrative analysis of metagenomics and metabolomics (BLCA patients, EXP group; healthy individuals, CON group). (**a**) Redundancy analysis (RDA) at the species level. Dots of different colors or shapes indicate different samples. Arrows indicate metabolite information. A projection was made from the point to the arrow of the metabolite, and the distance of the projection point from the origin represents the relative influence of the metabolite on the distribution of the sample community. (**b**) Heat map of correlation coefficients between significantly different microbiomes and metabolites in two groups. Each row indicates a microbiome at the species level. Each column indicates a significantly different metabolite. The correlation coefficient R is expressed in color. R > 0 indicates positive correlation (red); R < 0 indicates negative correlation (blue). * indicates *p*-value < 0.05; ** indicates *p*-value < 0.01; *** indicates *p*-value < 0.001.

In order to select the relevant microbes and metabolites for the network analysis, data were further processed. First, we performed redundancy analysis (RDA) to reflect the relationship between gut microbiomes and environmental factors (significant fecal metabolites). As shown in Figure 7a, after filtering, the effects of the top 11 metabolites with the most significant differences (metabolites with top 11 VIP values and *p*-value < 0.05 with qualitative information) on species data were visualized at the species level. Next, the correlation coefficients between significantly different microbiomes and metabolites in two groups were calculated using Spearman analysis and presented in a cluster heat map. As shown in Figure 7b, the results showed that *Clostridium_sp__CAG_590* was positively related to various metabolites with statistical significances, such as 11Z-eicosenoic acid, arachidic acid, conjugated linoleic acids, elaidic acid, linoelaidic acid, linoleic acid, oleic acid, and petroselinic acid. However, *Bacteroides_salyersiae* was negatively related to these metabolites. Moreover, *uncultured_Bacteroides_sp* was significantly negatively related to ricinoleic acid. Furthermore, a hydroxy fatty acid, cholesterol sulfate, was positively related to *Bacteroides_fragilis*, *Parabacteroides_distasonis*, and *Bacteroides_eggerthii* with statistical significances. These results offered us many possibilities for the verification tests of these networks in BLCA.

## 4. Discussion

In the present study, to identify and analyze the differences of the gut microbiota and metabolites in BLCA, we characterized the genic, microbial, and functional repertoire and targeted metabolites of the microbiomes using metagenomics and metabolomics in fecal samples of 32 BLCA patients and 15 gender-, age-, and body-weight-matched healthy controls. Because of the role of gut microbiota in shaping the final fecal metabolome, we performed the combined study of both metagenomics and metabolomics data that could help us explore the potential biomarkers and molecular mechanisms in the future. Finally, the results showed that some special gut microbiomes and their key metabolites were significantly increased or decreased in BLCA patients compared to controls, which indicated a dysbiosis in BLCA patients. These findings provided great possibilities to explore the positive association between intestinal dysbiosis and BLCA. To our knowledge, this is the first human study using fecal metagenomics and metabolomics conducted in BLCA.

In fact, the gut has a large number of microorganisms—about 80% of the body’s normal microorganisms are concentrated here, and they are known as the body’s second largest genome. The gut microbiota is important for maintaining intestinal metabolism, intestinal homeostasis, and the intestinal immune system. Therefore, gut microbiota directly or indirectly affects the tumor progression [23]. Especially in colorectal cancer [24], the positive association between intestinal dysbiosis and colorectal cancer is well established. This is because feces are proximal to the colorectal mucosa. In terms of mechanism, TJALSMA [25] proposed a “driver–passenger” model of gut microbiota carcinogenesis, suggesting that certain specific intestinal bacteria can induce DNA damage in intestinal epithelial cells, leading to the initiation of carcinogenesis. These microorganisms are called “drivers”. In the process of carcinogenesis, the intestinal microenvironment is changed; certain conditionally pathogenic bacteria proliferate preferentially; and the “driver” microorganisms are gradually replaced by these “passenger” microorganisms, thus promoting tumor progression. For BLCA, during the process, inflammation, immunity, and microbial dysbiosis are probable contributors to the known increase in cancer incidence [26]. For example, one indicator of systemic inflammation, serum neutrophil/lymphocyte ratio (NLR), was predictive of disease recurrence and progression in both NMIBC and MIBC [27]. Moreover, in another study, ROS concentration correlated positively with increased tumorigenesis in mice treated with the carcinogen N-butyl-N-(4-hydroxybutyl) nitrosamine (BBN) and intravesical LPS compared with mice treated with BBN alone [28]. These support the idea that dysregulated inflammation and immunity are closely related to BLCA progression. In terms of gut microbiome dysbiosis and BLCA, to date, only one human study has been conducted [29]. Using pyrosequencing and the real-time quantitative polymerase chain reaction (PCR) method, the authors found a reduced content of *Clostridium* cluster XI and *Prevotella* in BLCA patients after comparisons in a study including 26 BLCA patients and 16 age-matched healthy participants. Moreover, the quantities of fecal butyric acid were found to be significantly decreased in BLCA patients. In our study, using metagenomic sequencing, we listed the top 35 microbiomes in abundance for two groups. The results showed not only *Clostridium* and *Prevotella*, but also 19 microbiomes including *Lactobacillus*, *Bifidobacterium*, and *Roseburia* were significantly reduced in fecal samples of BLCA patients at the genus level. Our findings were in line with the previous study [29]. Moreover, in our study, we also found some probiotics were reduced in BLCA patients such as *Lactobacillus*, *Bifidobacterium*, and *Butyrate-producing bacterium*, which were demonstrated to play a key role in protecting the body and inhibiting the tumor growth. For example, in Japan, the gut microbiome was reported to alter the NMIBC recurrence because of widely consumed *Lactobacillus* in fermented milk products and the reported anti-tumor properties of oral *Lactobacillus* preparations in mice [30]. A study reported that an oral cancer vaccine using a Bifidobacterium vector could suppress tumor growth in a syngeneic mouse BLCA model. Moreover, they found oral cancer vaccine alone or as an adjunct to anti-PD-1 antibody could provide a novel treatment option for patients with BLCA [31]. For *Butyrate-producing bacterium*, such as *Roseburia*, which was reduced in BLCA patients in our study, was found to act as probiotic for restoration of beneficial flora, thus reducing a major structural imbalance of gut microbiota in cancer patients [32].

An important mechanism of gut microbiota is through the multiple metabolites that may influence tumorigenic progression by regulating apoptosis, proliferation, inflammation, and immunity. SCFAs, produced by the gut microbiome, are major energy sources for enterocytes and are ligands for G protein-coupled free fatty acid receptors, being able to repress the histone deacetylase, which can directly limit tumor progression via prevention of pro-tumorigenic epigenetic changes [33]. For example, in prostate cancer, gut microbiota could promote tumor proliferation through regulating systemic and local prostate IGF1 in the host, which was mediated by SCFAs [13]. Moreover, secondary bile acids (BAs) are another type of bacterial metabolite, such as deoxycholic acid (DCA) and lithocholic acid (LCA), which were identified as acting as risk factors for colonic inflammation and cancer [34]. One study demonstrated gut-microbiome-mediated primary-to-BA conversion could regulate liver antitumor immunosurveillance [35]. In the metabolomics section of our study, we found that some SCFAs (propionic acid and butyric acid) were reduced, and BAs (DCA and LCA) were increased in BCLA patients, despite statistical significance not being obtained (*p*-value > 0.05). We speculated that the sample size may interfere the statistical calculations. Notably, we have some interesting findings that some long chain fatty acids (LCFAs) were significantly reduced in fecal samples of BLCA patients, including 11Z-eicosenoic acid, elaidic acid, petroselinic acid, ricinoleic acid, oleic acid, and arachidic acid. The study found that the production of C18-3OH by bacteria could be one of the mechanisms implicated in the anti-inflammatory properties of probiotics. The production of LCFA-3OH could be implicated in the microbiota/host interactions [36]. In addition, LCFAs produced by gut microbiota could form a synergistic triad with the immune system and regulate inflammation to maintain homeostasis, thus preventing cancer progression related to inflammation [37]. Therefore, we reasonably speculate that not only SCFAs but also LCFAs play important roles in preventing BLCA progression. One probable mechanism might be inflammation regulation and intestinal barrier function protection [16].

Next, in terms of integrated analysis of metagenomics and metabolomics, two points should be focused on. First, the results of our study showed *Clostridium_sp__CAG_590* was positively related to various metabolites with statistical significances, such as 11Z-eicosenoic acid, arachidic acid, conjugated linoleic acids, elaidic acid, linoelaidic acid, linoleic acid, oleic acid, and petroselinic acid. This microbiome and these metabolites were both reduced in BLCA patients after sequencing. These metabolites belong to LCFAs and lineolic acids with derivatives. One study regarding inflammatory bowel disease demonstrated that LCFAs (such as arachidic and oleic acids) and *Clostridium* were decreased in patients, which indicated an interaction network to identify key micronutrients, microbiota components, and metabolites that contribute to inflammatory bowel disease [38]. Moreover, in pediatric Crohn’s disease, higher abundances of *Clostridium* and linoleic acid were closely associated with a sustained response in patients [39]. Thus, we speculated that decreased abundances of *Clostridium* might positively regulate LCFAs and lineolic acids, thus acting as regulatory roles in BLCA progression via regulating inflammation. Notably, cholesterol sulfate was positively related to *Bacteroides fragilis*, *Parabacteroides distasonis*, and *Bacteroides_eggerthii* with statistical significances. Recently, researchers discovered that *Bacteroides* spp. was the specific bacteria metabolizing cholesterol for the first time [40]. Moreover, in primary mammary fat pad tumors, cholesterol sulfate was significantly increased in lung metastases, which suggests potential extracellular uptake by cancer cells [41]. Cholesterol sulfate was produced by hydroxysteroid sulfotransferase 2B1 (SULT2B1) and accumulates in human prostate adenocarcinoma and precancerous prostatic intraepithelial neoplasia (PIN) lesions, which indicates the functional roles of cholesterol sulfate in these diseases [42]. Therefore, further study focusing on the network of cholesterol sulfate and key microbiomes could be conducted.

However, this study had several limitations. First, it is necessary to expand the UBC tissue samples in order to explore the possibilities of specific microbiomes and metabolites as biomarkers for UBC detection or follow-up. Next, the present study integrated metabolomics and microbiome data of BLCA. However, the verifications of networks of specific microbiomes and metabolites and their regulatory roles in BLCA should be conducted in our future study. Last but not least, it could be quite interesting to explore differences in gut microbiota and their metabolites on the basis of different pathological characteristics of BLCA. The readers must know that biological replicates (multiple extractions of the same fecal sample) would be more informative rather than a single data point for the metagenome that is known to be highly variable.

## 5. Conclusions

In summary, using metagenomics and metabolomics in BLCA in a Chinese population, we identified the alterations of gut microbiomes including *Streptococcus*, *Blautia*, *Bifidobacterium*, *Lactobacillus*, and *Eubacterium*. We also found altered metabolites including 11Z-eicosenoic acid, 3-methoxytyrosine, abrine, aniline-2-sulfonate, arachidic acid, conjugated linoleic acids, elaidic acid, glycylleucine, glycylproline, leucyl-glycine, linoelaidic acid, linoleic acid, nicotinamide hypoxanthine dinucleotide, oleic acid, petroselinic acid, ricinoleic acid, and cholesterol sulfate. Moreover, using integrated analysis, we preliminarily revealed the associations between specific gut microbiomes and metabolites, such as *Bacteroides_salyersiae* and some long chain fatty acids. These findings determined potential causative links among gut dysbiosis, dysregulated metabolites, and BLCA.

## Data Availability

The data presented in this study are available on request from the corresponding author. The data are not publicly available due to institutional policy.

## Supplementary Materials

The following supporting information can be downloaded at https://www.mdpi.com/article/10.3390/genes13111967/s1, Figure S1: The KEGG pathway maps with statistical significances. Table S1: List of top 35 microbiomes in terms of abundance. Table S2: List of functional characterization of gut microbiota. Table S3: The KEGG pathway annotation results.

## Abbreviations

BLCA: bladder cancer; UBC, urothelial bladder carcinoma; MIBC, muscle-invasive bladder cancer; NMIBC, non-muscle-invasive bladder cancer; SCFA, short-chain fatty acid; PCoA, principal co-ordinates analysis; NMDS, non-metric multi-dimensional scaling; LDA, linear discriminant analysis; KEGG, Kyoto Encyclopedia of Genes and Genomes; eggnog, Evolutionary Genealogy of Genes: Non-Supervised Orthologous Groups; CAZy, Carbohydrate-Active Enzymes Database; CTA, chemical taxonomy attribution; RDA, redundancy analysis; NLR, neutrophil/lymphocyte ratio; BBN, N-butyl-N-(4-hydroxybutyl) nitrosamine; PCR, polymerase chain reaction; BA, bile acid; DCA, deoxycholic acid; LCA, lithocholic acid; LCFA, long-chain fatty acid; SULT2B1, sulfotransferase 2B1; PIN, prostatic intraepithelial neoplasia; PCA, Pareto-scaled principal component analysis; FDR, false discovery rate; OPLS-DA, orthogonal partial least-squares discriminant analysis; VIP, variable importance value.
