# Supplementary material for: Integrated Analysis of the Fecal Metagenome and Metabolome in Bladder Cancer in a Chinese Population
Source: Genes (Basel). 2022 Oct 28;13(11):1967. doi: 10.3390/genes13111967 (PMC9690037; doi:10.3390/genes13111967)
Supplement: Supplementary file 1 [file genes-13-01967-s001.zip › genes-1968801-supplementary.pdf]

Supplementary Material

Integrative Analysis of Fecal Metagenomics and Metabolomics in Bladder Cancer in a Chinese Population

Chuan Qin, Zhenghao Chen, Rui Cao, Mingjun Shi and Ye Tian *

Department of Urology, Beijing Friendship Hospital, Capital Medical University, Beijing 100068, China
* Correspondence: youyitianye@126.com; Tel.: +86-010-63138377

Table S1. List of top 35 microbiomes in terms of abundance.

Genus	Abundance trend
Parabacteroides	Higher in EXP
Butyricicoccus	Higher in EXP
Megamonas	Higher in CON
Prevotella	Higher in CON
Fusobacterium	Higher in EXP
Eubacterium	Higher in CON
Dialister	Higher in CON
Veillonella	Higher in EXP
Escherichia	Higher in EXP
Anaerostipes	Higher in CON
Streptococcus	Higher in EXP
Fusicatenibacter	Higher in CON

Bacteroides	Higher in EXP
Oscillibacter	Higher in CON
Lactobacillus	Higher in CON
Roseburia	Higher in CON
Megasphaera	Higher in CON
Gemmiger	Higher in CON
Ruminococcus	Higher in EXP
Phascolarctobacterium	Higher in EXP
Sutterella	Higher in CON
Romboutsia	Higher in CON
Faecalibacterium	Higher in CON
Bifidobacterium	Higher in CON
Coprococcus	Higher in CON
Paraprevotella	Higher in EXP
Collinsella	Higher in EXP
Clostridium	Higher in CON
Lachnoclostridium	Higher in EXP
Dorea	Higher in CON
Akkermansia	Higher in EXP
Alistipes	Higher in CON

Subdoligranulum	Higher in CON
Bilophila	Higher in EXP
Blautia	Higher in CON
**Species**	**Abundance trend**
Bacteroides fragilis	Higher in EXP
Bacteroides thetaiotaomicron	Higher in EXP
Roseburia faecis	Higher in CON
Roseburia inulinivorans	Higher in CON
Faecalibacterium prausnitzii	Higher in CON
[Eubacterium] hallii	Higher in CON
Escherichia coli	Higher in EXP
Blautia wexlerae	Higher in CON
[Ruminococcus] torques	Higher in CON
[Eubacterium] rectale	Higher in EXP
Bifidobacterium adolescentis	Higher in CON
Bacteroides vulgatus	Higher in EXP
uncultured Ruminococcus sp.	Higher in CON
Blautia obeum	Higher in CON
Clostridium sp. CAG:7	Higher in EXP
uncultured Clostridium sp.	Higher in CON

Bifidobacterium longum	Higher in EXP
Dialister succinatiphilus	Higher in CON
Alistipes putredinis	Higher in CON
Bacteroides stercoris	Higher in EXP
Roseburia intestinalis	Higher in CON
Prevotella copri CAG:164	Higher in CON
Bacteroides ovatus	Higher in CON
Fusicatenibacter saccharivorans	Higher in CON
Akkermansia sp. CAG:344	Higher in EXP
Dorea longicatena	Higher in CON
Ruminococcus callidus	Higher in EXP
Bifidobacterium pseudocatenulatum	Higher in CON
Megamonas funiformis	Higher in CON
Bacteroides plebeius	Higher in EXP
Bacteroides dorei	Higher in EXP
Bacteroides coprocola	Higher in CON
Bacteroides uniformis	Higher in EXP
Prevotella copri	Higher in CON
Eubacterium sp. CAG:38	Higher in EXP

BLCA patients, EXP group; healthy individuals, CON group.

Table S2. List of functional characterization of gut microbiota.

KEGG pathway	EXP relative abundance	CON relative abundance
Organismal Systems; Immune system	0.000928	0.000973
Genetic Information Processing; Folding, sorting and degradation	0.008625	0.008780
Human Diseases; Cancers: Overview	0.002075	0.002042
Organismal Systems; Nervous system	0.000725	0.000660
Metabolism; Lipid metabolism	0.009825	0.009856
Human Diseases; Endocrine and metabolic diseases	0.001326	0.001403
Metabolism; Nucleotide metabolism	0.017829	0.018526
Human Diseases; Cardiovascular diseases	0.001040	0.001101
Cellular Processes; Cellular community - prokaryotes	0.011554	0.011921
Genetic Information Processing; Replication and repair	0.011273	0.011770
Cellular Processes; Transport and catabolism	0.002157	0.001922
Genetic Information Processing; Translation	0.019353	0.020376
Organismal Systems; Environmental adaptation	0.000752	0.000733

Metabolism; Energy metabolism	0.019885	0.020447
Cellular Processes; Cell motility	0.002126	0.002373
Organismal Systems; Aging	0.001272	0.001327
Genetic Information Processing; Transcription	0.001031	0.001072
Metabolism; Metabolism of cofactors and vitamins	0.023138	0.023457
Environmental Information Processing; Signal transduction	0.009662	0.009675
Human Diseases; Neurodegenerative diseases	0.000403	0.000365
Metabolism; Glycan biosynthesis and metabolism	0.010453	0.010184
Metabolism; Carbohydrate metabolism	0.039645	0.040542
Organismal Systems; Digestive system	0.000498	0.000501
Human Diseases; Drug resistance: Antineoplastic	0.001292	0.001315
Human Diseases; Infectious diseases: Viral	0.000324	0.000320
Human Diseases; Drug resistance: Antimicrobial	0.005727	0.005662
Metabolism; Biosynthesis of other secondary metabolites	0.007913	0.007904
Metabolism; Metabolism of terpenoids and polyketides	0.005170	0.005238

Metabolism; Metabolism of other amino acids	0.008116	0.008037
Metabolism; Amino acid metabolism	0.030106	0.030568
Environmental Information Processing; Membrane transport	0.020228	0.021590
Human Diseases; Infectious diseases: Bacterial	0.002343	0.002357
Metabolism; Xenobiotics biodegradation and metabolism	0.003032	0.003175
Cellular Processes; Cell growth and death	0.004911	0.005015
Organismal Systems; Endocrine system	0.002282	0.002359

**eggNOG pathway**	**EXP relative abundance**	**CON relative abundance**
Resolvase	0.002667	0.003035
Transporter	0.002213	0.002163
Integrase	0.002746	0.002642
Binding-protein-dependent transport systems inner membrane component	0.002713	0.003045
transposase	0.007272	0.007228
oxidoreductase	0.001693	0.001642
peptidase_	0.002346	0.002177
Transposase	0.005297	0.006012
Glycosyl transferase (Group 1	0.001749	0.001475

ABC transporter (permease)	0.002362	0.002386
hydrolase family 43	0.001865	0.001684
radical SAM domain protein	0.002406	0.002516
Dehydrogenase	0.002745	0.002809
Hydrolase	0.002459	0.002451
helicase	0.002353	0.002495
AAA-ATPase	0.001627	0.001732
Ragb susd domain-containing protein	0.002919	0.002354
Transcriptional Regulator AraC Family	0.001952	0.001959
Glycosyl transferase, family 2	0.003903	0.003640
domain protein	0.005284	0.005281
membrane	0.001948	0.002067
Histidine kinase	0.011125	0.010771
Efflux transporter rnd family, mfp subunit	0.001896	0.001526
Membrane	0.007120	0.006665
acetyltransferase	0.003057	0.002800
hydrolase family 2	0.002524	0.002198
regulatoR	0.003367	0.003686
Methyltransferase	0.002679	0.002712

RNA Polymerase	0.002477	0.002141
ATPase (AAA	0.001599	0.001866
hydrolase family 3	0.001669	0.001634
TonB dependent receptor	0.004881	0.003875
Mate efflux family protein	0.003663	0.003927
tonB-dependent Receptor	0.001983	0.001705
Transcriptional regulator	0.011444	0.012176

**CAZy pathway**	**EXP relative abundance**	**CON relative abundance**
amylo-&alpha_-1,6-glucosidase (EC 3.2.1.33)	0.001253	0.001430
dTDP-L-Rha: N-acetylglucosaminyl-PP-decaprenol &alpha_-1,3-L-rhamnosyl(...)	0.002248	0.002122
branching enzyme (EC 2.4.1.18)	0.001253	0.001424
pullulanase (EC 3.2.1.41)	0.001150	0.001337
sucrose phosphorylase (EC 2.4.1.7)	0.001150	0.001337
chitin oligosaccharide synthase (EC 2.4.1.-)	0.002248	0.002122
N-acetylgalactosaminyltransferase (EC 2.4.1.-)	0.002248	0.002122
hyaluronan synthase (EC 2.4.1.212)	0.002248	0.002122
UDP-Galf: galactofuranosyl-galactofuranosyl-rhamnosyl-N-acetylglucosam	0.002248	0.002122

dolichyl-phosphate &beta_-glucosyltransferase (EC 2.4.1.117)	0.002248	0.002122
lysozyme (EC 3.2.1.17)	0.001510	0.001401
exo-&beta_-glucosaminidase (EC 3.2.1.165)	0.002537	0.002171
[inverting] exo-&alpha_-1,5-L-arabinanase (EC 3.2.1.-)	0.002385	0.002072
exo-1,3-1,4-glucanase (EC 3.2.1.-)	0.001401	0.001367
coniferin &beta_-glucosidase (EC 3.2.1.126)	0.001401	0.001367
dolichyl-phosphate &beta_-D-mannosyltransferase (EC 2.4.1.83)	0.002248	0.002122
isoprimeverose-producing oligoxyloglucan hydrolase (EC 3.2.1.120)	0.001401	0.001367
arabinanase (EC 3.2.1.99)	0.002385	0.002072
xylan 1,4-&beta_-xylosidase (EC 3.2.1.37)	0.001401	0.001367
exo-&alpha_-1,5-L-arabinofuranosidase (EC 3.2.1.-)	0.002385	0.002072
cellulose synthase (EC 2.4.1.12)	0.002248	0.002122
UDP-Galf: rhamnopyranosyl-N-acetylglucosaminyl-PP-decaprenol &beta_-1,(...)	0.002248	0.002122
xylanase (EC 3.2.1.8)	0.002477	0.002139
glucan 1,3-&beta_-glucosidase (EC 3.2.1.58)	0.001404	0.001369

N-acetylglucosaminyltransferase (EC 2.4.1.-)	0.002248	0.002122
neopullulanase (EC 3.2.1.135)	0.001150	0.001337
glucodextranase (EC 3.2.1.70)	0.001178	0.001356
acetyl xylan esterase (EC 3.1.1.72)	0.001510	0.001535
4-&alpha_-glucanotransferase (EC 2.4.1.25)	0.001253	0.001424
chitin synthase (EC 2.4.1.16)	0.002248	0.002122
galactan 1,3-&beta_-galactosidase (EC 3.2.1.145)	0.002385	0.002072
maltotriose-forming &alpha_-amylase (EC 3.2.1.116)	0.001150	0.001337
cyclomaltodextrinase (EC 3.2.1.54)	0.001253	0.001424
mannosylglycoprotein endo-&beta_-mannosidase (EC 3.2.1.152)	0.002151	0.001884
glucan 1,4-&beta_-glucosidase (EC 3.2.1.74)	0.001401	0.001367

BLCA patients, EXP group; healthy individuals, CON group.

Table S3. The KEGG pathway annotation results

**Map Name**	**Map ID**	**P value**	**FDR**	**RichFactor**
Biosynthesis of unsaturated fatty acids	hsa01040	0.006	0.031	0.027
Linoleic acid metabolism	hsa00591	0.046	0.115	0.036

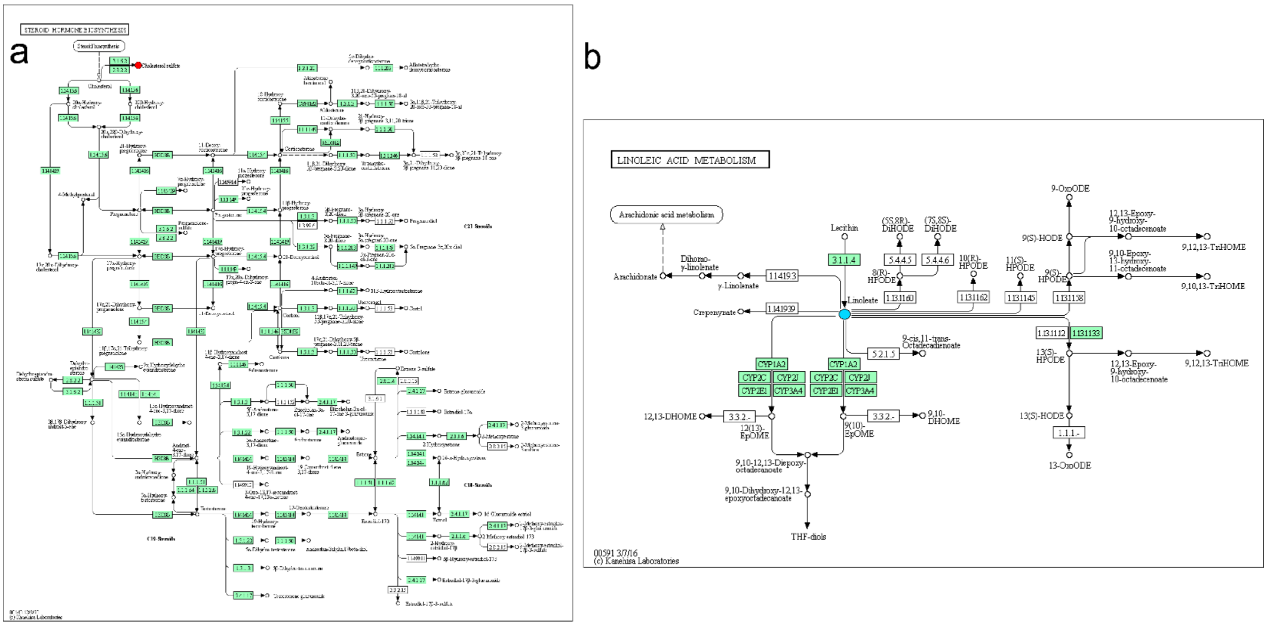

Figure S1. The KEGG pathway maps with statistical significances. (a) Biosynthesis of unsaturated fatty acids. (b) Linoleic acid metabolism. The small circles in the metabolic pathway diagram represented metabolites.
